# Acute stroke treatment and outcome in the oldest old (90 years and older) at a tertiary care medical centre in Germany-a retrospective study showing safety and efficacy in this particular patient population

**DOI:** 10.1186/s12877-021-02566-3

**Published:** 2021-10-29

**Authors:** Jil Kauffmann, Daniel Grün, Umut Yilmaz, Gudrun Wagenpfeil, Klaus Faßbender, Mathias Fousse, Marcus M. Unger

**Affiliations:** 1 Department of Neurology, Saarland University Medical Center, Kirrberger Str., 66421 Homburg, Germany; 2 Department of Neuroradiology, Saarland University Medical Center, Kirrberger Str., 66421 Homburg, Germany; 3 Institute of Medical Biometry, Epidemiology and Medical Informatics, Saarland University Medical Center, Kirrberger Str., 66421 Homburg, Germany

**Keywords:** Stroke, Geriatrics, Elderly, Thrombolysis, Thrombectomy

## Abstract

**Background:**

Stroke is among the most common causes of death and disability worldwide. Despite the relevance of stroke-related disease burden, which is constantly increasing due to the demographic change in industrialized countries with an ageing population and consecutively an increase in age-associated diseases, there is sparse evidence concerning acute stroke treatment and treatment-related outcome in the elderly patient group. This retrospective study aimed at analysing patient characteristics, therapy-related complications and functional outcome in stroke patients aged 90 years or older who underwent acute stroke treatment (i.e. intravenous thrombolysis, mechanical thrombectomy, or both).

**Methods:**

We identified files of all inpatient stays at the Department of Neurology at Saarland University Medical Center (tertiary care level with a comprehensive stroke unit) between June 2011 and December 2018 and filtered for subjects aged 90 years or older at the time of admission. We reviewed patient files for demographic data, symptoms upon admission, (main) diagnoses, comorbidities, and administered therapies. For patients admitted due to acute stroke we reviewed files for therapy-related complications and functional outcome. We compared the modified Rankin scale (mRS) scores upon admission and at discharge for these patients.

**Results:**

We identified 566 inpatient stays of subjects aged 90 years or older. Three hundred sixty-seven of the 566 patients (64.8%) were admitted and discharged due to symptoms indicative of stroke. Two hundred eleven patients received a diagnosis of ischaemic stroke. These 211 patients were analysed subsequently. Sixty-four patients qualified for acute stroke treatment (intravenous thrombolysis *n* = 22, mechanical thrombectomy *n* = 26, intravenous thrombolysis followed by mechanical thrombectomy *n* = 16) and showed a significant improvement in their functional status as measured by change in mRS score (admission vs. discharge, p 0.001) with 7 (10.9%) observed potentially therapy-related complications (relevant drop in haemoglobin *n* = 2, subarachnoidal haemorrhage *n* = 1, cerebral haemorrhage *n* = 3, extracranial bleeding n = 1). One intravenous thrombolysis was stopped because of an uncontrollable hypertensive crisis. Patients who did not qualify for these treatments (including those declining acute treatment) did not show a change of their functional status between admission and discharge (p 0.064).

**Conclusion:**

Our data indicate that acute stroke treatment is effective and safe in the oldest old. Age alone is no criterion to withhold an acute intervention even in oldest old stroke patients.

**Supplementary Information:**

The online version contains supplementary material available at 10.1186/s12877-021-02566-3.

## Background

Owing to the demographic change in industrialized countries, the health-related and socioeconomic burden of stroke is constantly increasing [1]. While there is good evidence for the effectiveness and safety of therapeutic interventions in mid to late adulthood patients presenting with acute stroke, only sparse data exist for older age groups. Most of the trials investigating therapeutic interventions in acute stroke (i.e. intravenous thrombolysis with recombinant tissue plasminogen activator [2, 3] and mechanical thrombectomy [4, 5]) did not enroll subjects above the age of 80 years. This is somehow surprising, as it is exactly this group which is particularly prone to developing stroke due to the wide array of age-associated comorbidities resulting in an elevated cerebrovascular risk profile [6, 7]. Only sparse data are available regarding specific treatments in stroke patients older than 80 years. Yet, existing data indicate that mechanical thrombectomy and intravenous thrombolysis are associated with a favourable outcome in this patient cohort as well [8, 9].

Taking into consideration that physiological age-related changes make elderly subjects respond differently to therapeutic interventions, evidence gained in younger patient groups with acute stroke cannot directly be transferred to geriatric patients.

This paucity of evidence consecutively leads to a lack of acute stroke treatment recommendations regarding geriatric patients and potentially exposes these patients to suboptimal care.

The primary goal of the present study was therefore analysing the clinical characteristics, administered therapeutic interventions, therapy-related complications and the functional outcome of the oldest old patients (aged 90 years and older) admitted due to acute stroke to our department, a tertiary care medical center with a comprehensive stroke unit. Moreover, we also aimed at identifying factors preventing these patients from receiving a potentially disability-reducing treatment.

## Methods

This study was approved by the local IRB (Ethikkommission der Aerztekammer des Saarlandes, Faktoreistrasse 4, 66111 Saarbruecken, Germany). The registration number of the study is 82/19. Due to the retrospective study design and the use of only anonymised data, the need for informed consent was waived by the IRB. The protocol was performed in accordance with the relevant local guidelines and regulations, including regulations on data protection. Anonymised data were retrieved from patients’ electronic medical files.

**Fig. 1:**
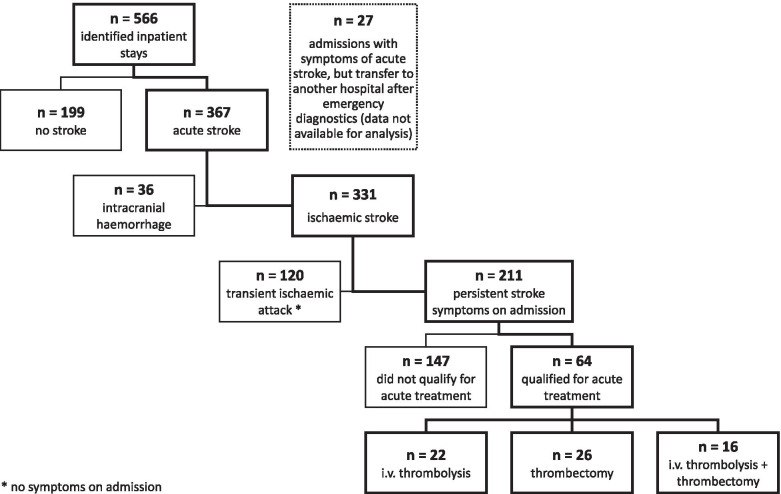
Flow chart visualizing identification of patients that were subsequently analysed

In a first step, we identified all emergency admissions to the Department of Neurology of the Saarland University Medical Center between June 2011 and December 2018 that resulted in an in-patient hospital stay. Hereafter, we selected all patients above the age of 90 and classified cases based on the discharge diagnosis as either admissions due to acute stroke or other conditions. For non-stroke cases, basic demographic data and diagnoses were analysed. Patients with acute stroke were grouped into one of the three following categories: a) patients with intracranial haemorrhage, b) patients diagnosed at discharge with a transient ischaemic attack (TIA) and c) patients with a diagnosis of ischaemic stroke. For the latter group, we checked whether or not an acute therapeutic intervention (i.e. intravenous thrombolysis with recombinant tissue plasminogen activator (rtPA), mechanical thrombectomy or both) had been performed (see flow diagram Fig. 1). To categorize the subtypes of ischaemic stroke based on aetiology, we referred to the Trial of Org 10172 in Acute Stroke Treatment (TOAST) classification [10] and added a category named “mixed aetioloy” if more than one plausible aetiology was identified in a patient. We also collected the following clinical data: main diagnosis, comorbidities, brain imaging, type of acute therapeutic intervention, TICI score (if applicable), door-to-needle and door-to-groin time (if applicable), therapy-related complications, NIHSS (National Institute of Health Stroke Scale) score [11], modified Rankin score (mRS) [12] on admission and at discharge, duration of hospital stay and mode of discharge (i.e. home, nursing facility, other hospital etc.). As the above mentioned main parameters of this study are mandatory for the certification of stroke units in Germany, these parameters were available for all analyzed subjects. For comorbidities and risk factors the number of missing values is given in Table 2.

For the analysed study period, all patients had been treated according to the same standard operating procedures in regard to their diagnosis and stroke treatment as previously described [13].

Statistics were carried out using IBM SPSS statistics version 25.0 (SPSS Inc., Chicago, IL, USA). The significance threshold was set at a *p* value < 0.05. To determine a significant relationship between nominal variables the Chi-squared test was used. The Mann-Whitney-U test was used to analyse differences in quantitative variables for two independent groups. To compare functional outcome (i.e. mRS score upon admission versus mRS score at discharge) within each of the two groups, we used the McNemar test. A functional status was defined as good for a mRS score of ≤2 and as poor if the mRS was > 2.

## Results

We identified 566 cases aged 90 years or older treated during the investigated 7½-year period. The median age in the total cohort was 91 years (range 90 to 102 years), median duration of inpatient stay was 5 days (range 1 to 40 days), 71% patients (399 of 566) were female.

### Cases admitted due to conditions other than stroke

One hundred ninety-nine cases were referred to our department due to conditions other than stroke. Categories for main diagnoses of these patients are listed in Additional Table 1a.

### Cases admitted due to symptoms indicating acute stroke

Most patients aged 90 years or older (64.8%, 367 of 566 respectively) were referred to our department due to symptoms indicating acute stroke. These patients were analyzed subsequently. The main focus of this study was on patients with ischaemic stroke. Demographic and clinical data of these patients are summarised in Additional Table 1b.

Thirty-six of 367 (9.8%) stroke patients showed cerebral haemorrhage on the computed tomography-scan (CT). In 30 of the 36 cases (83.3%) hemorrhage was intracerebral, 3 cases (8.3%) showed subarachnoidal, and 2 cases (5.6%) subdural haemorrhage. One patient showed haemorrhage in more than one space.

One hundred twenty patients (32.7%) of all patients admitted due to acute stroke received a diagnosis of transient ischaemic attack (TIA). The remaining 211 patients (57.5%) with persistent stroke symptoms and/or acute lesions in cerebral imaging were categorized as either receiving an acute therapeutic intervention for stroke (i.e. intravenous thrombolysis with recombinant tissue plasminogen activator (10.4%), mechanical thrombectomy (12.3%) or both (7.6%) or not receiving such an intervention. Demographic and clinical data of these two patient groups with acute stroke are summarised in Table 1. No difference existed between these two groups in regard to comorbidities (Table 2). Patients receiving an acute therapeutic intervention were more likely to have higher NIHSS scores (*p* < 0.001) and higher mRS scores upon admission (p < 0.001) as shown in Table 1.

**Table 1:** Demographic and clinical data of ischaemic stroke patients with and without an acute therapeutic intervention

	Cases receiving acute therapeutic intervention (*n* = 64)	Cases not receiving acute therapeutic intervention (*n* = 147)	*p* value
Age in years (median, [range])	92 [90–98]	91 [90–98]	0.906
Sex female / male (n, [%])	43 [67.2%] / 21 [32.8%]	111 [75.5%] / 36 [24.5%]	0.211
NIHSS score upon admission (median, [range])	14 [2–40]	6 [0–40]	< 0.001
mRS upon admission (median, [range])	5 [2–5]	3 [0–5]	< 0.001
Duration of inpatient stay in days (median, [range])	6 [2–40]	6 [1–23]	0.860
Number of drugs at the time of admission (median, [range])	6 [0–12]	6 [0–17]	0.533
Way of admission (n, [%])			0.001
1. Self-initiated 2. emergency service (paramedics)	- 11 [17.2%]	5 [3.4%] 67 [45.6%]	
3. emergency service (physician) 4. emergency room 5. other hospital	49 [76.6%] - 4 [6.2%]	5 [3.4%] 10 [6.8%] -	
Aetiology (n, [%])			0.357
1. large-artery atherosclerosis	32 [50.0%]	60 [40.8%]	
2. cardioembolism	10 [15.6%]	33 [22.4%]	
3. small-vessel occlusion	1 [1.6%]	10 [6.8%]	
4. stroke of other determined aetiology	–	–	
5. stroke of undetermined aetiology (at discharge)	18 [28.1%]	37 [25.2%]	
6. mixed aetiology	3 [4.7%]	7 [4.8%]	
Vessel occlusion (n, [%])			< 0.001
1. Large vessel occlusion (anterior circulation)	32 [50.1%]	17 [11.6%]	
2. Small vessel occlusion (anterior circulation)	13 [20.3%]	14 [9.5%]	
3. posterior circulation 4. none	6 [9.4%] 13 [20.3%]	5 [3,4%] 111 [75.5%]

**Table 2:** Comorbidities and risk factors in patients with acute ischaemic stroke

	Cases receiving acute therapeutic intervention (n = 64)	Cases not receiving acute therapeutic intervention (n = 147)	p value
**Hypertension** (n, [%]) missing values (n, [%])	58 (90.6%) 1 (1.6%)	125 (85.0%) 1 (0.7%)	0.195
**Atrial fibrillation** (n, [%]) missing values (n, [%])	37 (57.8%) 2 (3.1%)	65 (44.2%) 6 (4.1%)	0.054
**Dyslipidemia** (n, [%]) missing values (n, [%])	10 (15.6%) 1 (1.6%)	21 (14.3%) 1 (0.7%)	0.781
**Diabetes mellitus** (n, [%]) missing values (n, [%])	14 (21.9%) 1 (1.6%)	35 (23.8%) 1 (0.7%)	0.784
**History of stroke** (n, [%]) missing values (n, [%])	29(45.3%) 0	77 (42.3%) 0	0.345
**Smoking** (n, [%]) missing values (n, [%])	4 (6.3%) 1 (1.6%)	3 (2.1%) 1 (0.7%)	0.117

Of the 211 cases with ischemic stroke, 88 vessel occlusions were confirmed by CT-angiography at the time of admission.

The M1-segment of the middle cerebral artery (*n* = 27) was the most common location of vessel occlusion, followed by the M2-segment (*n* = 25) and the internal carotid artery (ACI, *n* = 18). The most frequent reason for not proceeding with a mechanical recanalisation was a prolonged time window (symptoms onset to admission): 20 patients have already showed signs of ischaemia on CT scans, 7 patients were admitted with an unknown time of symptoms onset.

Eight patients showed an occlusion of the basilar artery; 2 of these 8 patients underwent solely mechanical thrombectomy, 4 were treated with rtPA in addition to a mechanical thrombectomy, the remaining 2 patients did not receive an acute intervention (1 case due to marked stroke on the CT-scan upon admission, 1 case due to a restricted medical care regime reported in the patient’s living will).

Sixty-four (30.3%) of the 211 patients admitted with stroke symptoms underwent acute stroke treatment. The most frequent reasons for withholding an acute therapeutic intervention were: signs of manifest ischaemia on CT scan (27.5%), unknown or prolonged time since onset of symptoms (24.7%), spontaneous improvement of symptoms at the time of admission (4.7%) and anticoagulant therapy (6.6%). Clinical data of these subgroups are demonstrated in Table 3.

**Table 3:** Clinical data of all patients with ischaemic stroke having received an acute therapeutic intervention

	Cases receiving intravenous thrombolysis with recombinant tissue plasminogen activator (*n* = 38)	Cases receiving mechanical thrombectomy (*n* = 42)
Time onset-to-admission (n, [%])		
1. < 1 h	10 [26.3%]	12 [38.1%]
2. 1–2 h	15 [39.5%]	11 [26.2%]
3. 2–3 h	7 [18.4%]	3 [7.1%]
4. 3–4.5 h	2 [5.3%]	4 [9.5%]
5. > 4.5 h	2 [5.3%]	8 [19.0%]
6. unknown	2 [5.3%]	4 [9.5%]
Door-to-needle (n, [%])		
1. < 30 min	9 [23.7%]	
2. 30–45 min	4 [10.5%]	
3. 45–60 min	10 [26.3%]	
4. 60–120 min	5 [13.2%]	
5. > 120 min	2 [5.3%]	
6. before admission	5 [13.2%]	
7. unknown	3 [7.9%]	
Door-to-puncture (n, [%])		
1. 45–60 min		4 [9.5%]
2. 60–90 min		11 [26.2%]
3. > 90 min		3 [7.2%]
4. unknown		24 [57.1%]
Recanalisation score, TICI (n, [%])		
1. TICI 0		5 [11.9%]
2.TICI 1		1 [2.4%]
3.TICI 2a		1 [2.4%]
4.TICI 2b +2c		15 [35.7%]
5. TICI 3		20 [47.6%]

Thirty-eight patients received intravenous thrombolysis with rtPA. Subsequently, 16 of these 38 patients also underwent an attempt for mechanical thrombectomy due to large vessel occlusion. Two of these 16 patients with intravenous thrombolysis and a subsequent attempt for mechanical thrombectomy showed a spontaneous recanalisation without the need for mechanical thrombus extraction. 26 patients underwent an attempt for mechanical recanalisation without preceding intravenous thrombolysis with rtPA therapy. One of these 26 patients showed a spontaneous recanalisation without the need for mechanical thrombus extraction. With regard to the effectiveness of the mechanical thrombectomy, 29 cases of the 42 cases (i.e. 83.3%) who underwent an attempt for mechanical thrombectomy reached a TICI score of TICI 2b (defined as complete filling of all of the expected vascular territory but the filling slower than normal) or TICI 3 (complete reperfusion). Intervention-associated data of patients receiving acute stroke treatment are summarized in Table 3.

### Treatment-related complications and intra-hospital mortality

Secondary intracranial haemorrhage, as a potential complication of an acute stroke intervention, was diagnosed using CT scan imaging, routinely performed 24 h after the acute stroke intervention and at any time during the inpatient stay if the patient developed new neurological symptoms. Secondary intracranial haemorrhage was observed in 2 patients who underwent intravenous thrombolysis (5.3%), in 2 patients of the patients that underwent mechanical thrombectomy (4.8%) and in 2 patients without any acute intervention (1.4%).

Extracranial bleeding (gastrointestinal haemorrhage) was observed in 1 patient after intravenous thrombolysis (2.6%). Two patients (4.8%) showed a clinically relevant drop in haemoglobin (without evidence of a specific bleeding localization) after mechanical thrombectomy. Arterial dissection as a potential complication of mechanical thrombectomy was not observed in our cohort. Eight of the 64 patients (12.5%) who received an acute stroke intervention and 16 of the 147 patients (10.9%) who did not receive an acute stroke intervention died during the inpatient stay. There was no significant difference in intra-hospital mortality within the acute stroke treatment group (i.e. between patients receiving thrombectomy and patients receiving intravenous thrombolysis p 0.734) and no significant difference between the cohort receiving an acute stroke treatment and the cohort not receiving acute stroke treatment (p 0.661).

**Fig. 2:**
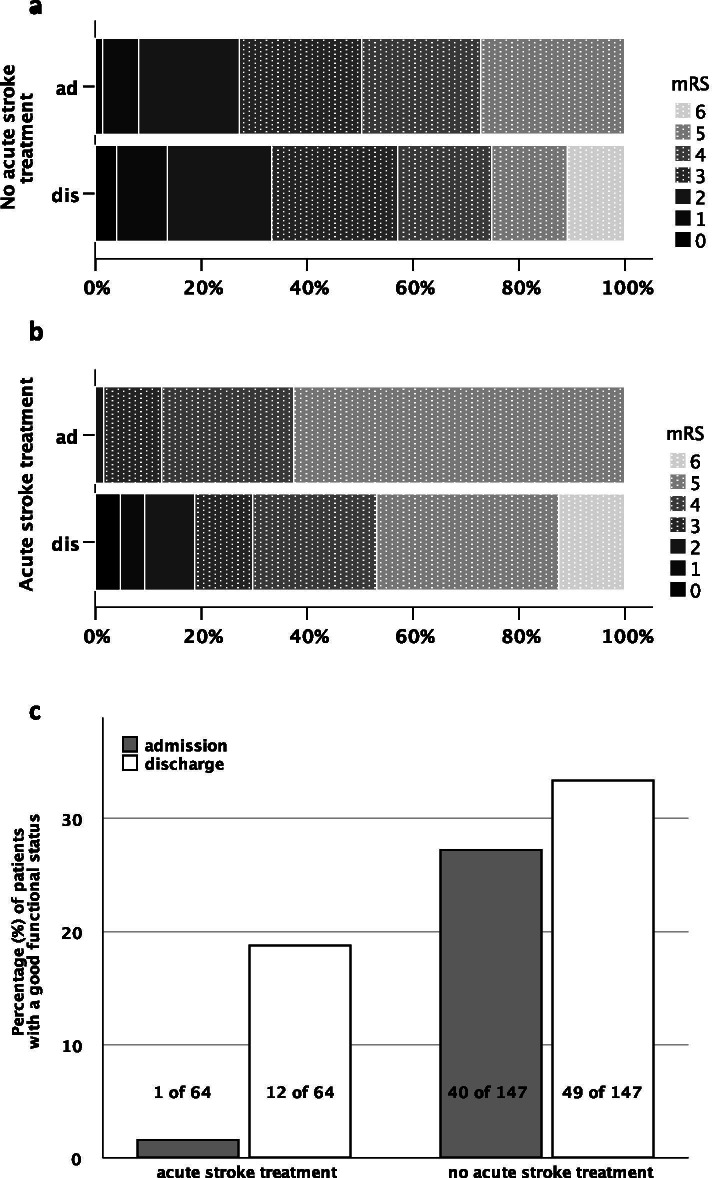
Distribution of modified Rankin Scale (mRS) scores upon admission (ad) and at discharge (dis). **a** shows the distribution for patients admitted due to acute stroke who did not qualify for or who declined acute stroke treatment („No acute stroke treatment“). **b** shows the distribution for patients admitted due to acute stroke who received intravenous thrombolysis, mechanical recanalisation or a combination of both („Acute stroke treatment“). **c** shows the absolute and relative (%) numbers of patients with a good function status (mRS ≤2) upon admission (grey bars) and at discharge (white bars) for both groups

To investigate whether our cohort of elderly patients enjoyed an improvement in their functional status (assessed by the modified Rankin Scale, mRS) after acute stroke treatment, we compared individual mRS scores upon admission to mRS scores at discharge. A good functional status was defined as a mRS score of ≤2, a poor functional status was defined as a mRS score > 2. Patients receiving an acute stroke treatment (either intravenous thrombolysis, mechanical recanalisation or both) showed a significant improvement in their functional status (admission versus discharge, p 0.001). For patients who did not qualify for or who declined such treatment, there was no significant change with regard to their functional status (admission versus discharge, p 0.064, Fig. 2). We also compared the group with acute stroke treatment to the group without acute stroke treatment. To this end, we determined patients with a poor functional status (mRS > 2) upon admission who converted to a good functional status (mRS ≤2) at discharge in each group. 17.2% (11 of 64) of the patients in the acute stroke treatment group and 9.5% (14/147) of the patients without acute stroke treatment converted to a good functional status at discharge. This descriptive difference in the rate of poor-to-good converters did not reach the level of statistical significance (p 0.113).

Concerning the mode of discharge (see Table 4), there was no significant difference between the different groups.

**Table 4:** Differences concerning the mode of discharge or transfer to another hospital between the acute therapeutic intervention group and the group without acute therapeutic intervention

	Cases receiving acute therapeutic intervention (***n*** = 64)	Cases not receiving acute therapeutic intervention (***n*** = 147)	p 0.917
**Community** (n, [%])	11 (17.2%)	31 (21.1%)	
**Rehabilitation facility** (n, [%])	9 (14.1%)	18 (12.2%)	
**Nursing home** (n, [%])	13 (20.4%)	33 (22.4%)	
**Internal medicine department** (n, [%])	21 (32.8%)	37 (25.2%)	
**Other neurological department** (n, [%])	2 (3.1%)	9 (6.1%)	
**Death** (n, [%])	8 (12.5%)	16 (10.9%)	
**Other departments** (n, [%])	0 (0%)	3 (2.1%)	

## Discussion

Our study investigated the safety and efficacy of acute stroke treatment in geriatric patients aged 90 years or older at a comprehensive stroke center.

Treatment related complications and treatment efficacy of acute stroke treatment (either intravenous thrombolysis, mechanical thrombectomy or both) have been intensively investigated. However, geriatric patients, especially nonagenarians, are underrepresented or even excluded in most relevant studies [14, 15]. Only few studies investigated acute stroke treatment in the oldest old for intravenous thrombolysis [16, 17] and mechanical thrombectomy [8, 18], leading to controversies regarding the applicability in this population and hence prompted us to investigate this controversial topic.

Due to the retrospective character of this study, all procedures (performed as routine clinical care) were uncontrolled. Yet, the same highly standardized operating procedures (SOPs) [13] applied to all patients admitted to our department during the investigated time period. Compliance with these SOPs in terms of streamlined diagnostic procedures and defined therapeutic pathways partially outweigh the lack of a study protocol (as it is used in prospective studies). Another strength of our study is that (in contrast to the majority of published studies) we analysed both therapeutic options of acute stroke treatment (intravenous thrombolysis and mechanical thrombectomy) over a representative period of time (7 ½ years). By this means we managed to extract data from 80 acute therapeutic interventions for stroke (38 thrombolysis, 42 mechanical thrombectomy) in 64 patients. To identify a potential selection bias, we also checked for admissions with symptoms indicating acute stroke that did not result in an in-patient stay at our hospital. In the investigated 7 ½ years only 27 patients aged 90 years or older who presented with symptoms indicating acute stroke were transferred to other hospitals after emergency diagnostics. None of these patients qualified for an acute stroke treatment. Hence, these 27 admissions that did not result in an in-patient stay at our hospital (as opposed to 367 admissions that did result in an in-patient stay) are unlikely to have a major effect on the reported results.

### Differences in the way of admission

We observed a significant difference in the way of admission between patients receiving an acute therapeutic intervention and those not receiving such a therapy. This difference was particularly pronounced for admissions by an emergency response physician (76.6% versus 3.4%). The more severe symptoms (in terms of higher mRS and NIHSS score) in the group that later received an acute therapeutic intervention might have triggered the admission by an emergency response physician.

### Comparison with published studies in terms of efficacy and safety

Concerning mechanical thrombectomy, successful recanalisation rates (defined as a mTICI score 2b or 3) after large vessel occlusion were described in 71% [4] in a meta-analysis of younger cohorts (median age 68, 57–77 years). Recanalisation rates for older cohorts show a high variability due to smaller sample sizes and differences in study design (selection of patients, selection of occluded vessels to be analysed, etc.). For patients aged 80 years or older, successful recanalisation is reported for 96% [19], 78.5% [8] and 54.2% [20]. For nonagenarians, successful recanalisation is reported for 79.3% [8] and 66.6% [21]. In the latter study, the recanalization rate in nonagenarians showed no significant difference compared to the recanalisation rate (62.3%) in patients younger than 90 years (with a mean age of 68.4 years). Hence, the recanalisation rate of 83.3% in our study is in accordance with those reported previously.

A meta-analysis of five randomized trials investigating mechanical thrombectomy reported a 90-day-mortality of 15.3% in patients with a median age of 68 years [4]. Yet, mortality is likely to be associated with age: Sussman and colleagues reported a 90-day-mortality of 40.9% for octogenarians and 63% for nonagenarians [8]. Due to the retrospective design of our analysis, only data on intra-hospital mortality are available for our cohort; intra-hospital mortality was 12.5%.

Intravenous thrombolysis for acute stroke has previously been reported being associated with a significant favourable functional outcome [9]. Intracranial haemorrhage following intravenous thrombolysis has been described in 2.5% among patients aged 80 years or older [9]. Yet, higher rates of intracranial haemorrhage have been reported in studies for nonagenarians receiving a combined therapy of mechanical thrombectomy and intravenous thrombolysis (e.g. 21.4% [8] and 33.3% [21]), compared with the rate of (symptomatic) intracranial haemorrhage of 4% in octogenarians [19] and 5% in younger patients [4]. The rate of intracranial haemorrhage in the reported cohort remained within the previously reported range for younger cohorts. Intracranial haemorrhage rates depend on the applied criteria e.g. the SITS-MOST definition [2] and reported intracranial haemorrhage rates are difficult to compare due to the lack of specification in reported results (symptomatic or asymptomatic) and the use of different radiographic classifications for post-thrombolysis haemorrhage [22]. Other described treatment-related complications like major access site issues [23] did not occur in our endovascular therapy cohort. All in all, we identified 11% treatment-related complications in our reported cohort.

Our data endorse the safety and efficacy of acute therapeutic interventions in stroke patients aged 90 years and older. Calendar age per se does not justify withholding these potent therapy options for the oldest old. As the time window between symptom onset and application of acute stroke treatment determines outcome, the time-is-brain-concept needs to be kept in mind when treating our oldest citizens, too. Indeed, the most frequent reason for not qualifying for an acute stroke intervention in our cohort was a protracted time window between onset of symptoms and admission to our hospital (52.2%). We compared the door-to-needle time between the reported 38 patients aged 90 years or older who received intravenous thrombolysis and all inpatients treated with intravenous thrombolysis for stroke in our hospital (*n* = 610, data not shown) in the investigated 7½-year-period. For the patients aged 90 years or older, only 23.7% had a door-to-needle time of less than 30 min, while 44.1% of all inpatients had a door-to-needle time of less than 30 min (data not shown). This difference might be partially explained by difficulties in obtaining medical history and establishing an intravenous line in this particular patient cohort. Yet, the lower percentage of geriatric patients with an optimal door-to-needle time indicates that there is still room for improvement concerning acute medical care for elderly stroke patients. We observed a similar difference for the door-to-groin time between patients aged 90 years or older (9.5% had a door-to-groin time of less than 1 h) and all inpatients that underwent mechanical thrombectomy (*n* = 292, 39.7% had a door-to-groin time of less than 1 h, data not shown).

With a worldwide ageing population at high risk for cerebrovascular diseases, our study highlights the necessity of focusing on this hitherto under-represented and neglected patient group in clinical trials. Acute stroke treatment with intravenous thrombolysis and/or mechanical thrombectomy is already recommended with high quality evidence for the younger generations [24] and, it should become the standard care option for geriatric patients as well.

The main limitations of our study are owed to the retrospective study design: a) the interventions were not controlled for and therapeutic decisions might have been biased by the treating physicians’ individual judgement; b) all analyses rely on the documented diagnoses at discharge; c) a selection bias concerning the admissions to our hospital (tertiary care medical centre with a comprehensive stroke unit) cannot be excluded and d) there is a lack of (90 day) follow-up data concerning functional outcome and mortality. With regard to the latter aspect, our data only reflect short-term outcome and mortality (during the intra-hospital phase). Nevertheless, our data can be considered as an approximation concerning overall safety and effectiveness.

## Conclusion

Our results show a significant improvement in the functional status (measured as change in mRS at the time of admission to mRS at discharge) in our geriatric cohort receiving intravenous thrombolysis, mechanical thrombectomy or both. The rates of treatment-related complications were comparable to those reported for younger age groups [4, 25–27]. Hence, our retrospective study indicates that acute therapeutic interventions in stroke are effective and safe in patients aged 90 years and older.

## Supplementary Information

**Additional file 1.** Additional Table 1a Categories of main diagnoses for patients admitted due to conditions other than stroke. Additional Table 1b Demographic and clinical data of all patients admitted due to acute stroke.

## Data Availability

The datasets used and/or analysed during the current study are available from the corresponding author on request.

## Abbreviations

ACI: Internal carotid artery
CT: Computed tomography-scan
mRS: Rankin score
NIHSS: National Institutes of Health Stroke Scale
rtPA: Recombinant tissue plasminogen activator
SOP: Standardised operating procedures
TIA: Transient ischaemic attack
TICI: Thrombolysis In Cerebral Infarction
TOAST: Trial of Org 10172 in Acute Stroke Treatment
