# Supplementary material for: Acute stroke treatment and outcome in the oldest old (90 years and older) at a tertiary care medical centre in Germany-a retrospective study showing safety and efficacy in this particular patient population
Source: BMC Geriatr. 2021 Oct 29;21:611. doi: 10.1186/s12877-021-02566-3 (PMC8556881; doi:10.1186/s12877-021-02566-3)
Supplement: Supplementary file 1 — Additional file 1. Additional Table 1a Categories of main diagnoses for patients admitted due to conditions other than stroke. Additional Table 1b Demographic and clinical data of all patients admitted due to acute stroke. [file 12877_2021_2566_MOESM1_ESM.docx]

**Additional Table 1a** Categories of main diagnoses for patients admitted due to conditions other than stroke.

|  | Cases with conditions other than stroke (n=199) |
| --- | --- |
| Cognitive decline, disturbance of consciousness, delirium | 127 (63.8%) |
| Conditions unrelated to the nervous system | 20 (10.1%) |
| Conditions of the nervous system unrelated to stroke | 19 (9.5%) |
| Pain | 18 (9.0%) |
| Infectious diseases, electrolyte imbalance, deterioration of general condition | 15 (7.5%) |

**Additional Table 1b** Demographic and clinical data of all patients admitted due to acute stroke.

|  | Cases with symptoms indicating acute stroke (n=367) |
| --- | --- |
| Age in years (median, [range]) | 91 [90 -99] |
| Sex female / male (n, [%]) | 262 [71.45 %] / 105 [28.6 %] |
| NIHSS score upon admission (median, [range]) | 4 [0-40] |
| Duration of inpatient stay in days (median, [range]) | 5 [1-40] |
